# Response of Pluripotent Stem Cells to Environmental Stress and Its Application for Directed Differentiation

**DOI:** 10.3390/biology10020084

**Published:** 2021-01-23

**Authors:** Taku Kaitsuka, Farzana Hakim

**Affiliations:** 1 School of Pharmacy at Fukuoka, International University of Health and Welfare, 137-1 Enokizu, Okawa, Fukuoka 831-8501, Japan; 2 Biogen Pharmaceutical Company, 225 Binney Street, Cambridge, MA 02142, USA; farzanahakimphd@gmail.com

**Keywords:** embryonic stem cells, induced pluripotent stem cells, stress response, hypoxic stress, oxidative stress, thermal stress, mechanical stress, differentiation

## Abstract

**Simple Summary:**

Environmental changes in oxygen concentration, temperature, and mechanical stimulation lead to the activation of specific transcriptional factors and induce the expression of each downstream gene. In general, these responses are protective machinery against such environmental stresses, while these transcriptional factors also regulate cell proliferation, differentiation, and organ development in mammals. In the case of pluripotent stem cells, similar response mechanisms normally work and sometimes stimulate the differentiation cues. Up to now, differentiation protocols utilizing such environmental stresses have been reported to obtain various types of somatic cells from pluripotent stem cells. Basically, environmental stresses as hypoxia (low oxygen), hyperoxia, (high oxygen) and mechanical stress from cell culture plates are relatively safer than chemicals and gene transfers, which affect the genome irreversibly. Therefore, protocols designed with such environments in mind could be useful for the technology development of cell therapy and regenerative medicine. In this manuscript, we summarize recent findings of environmental stress-induced differentiation protocols and discuss their mechanisms.

**Abstract:**

Pluripotent stem cells have unique characteristics compared to somatic cells. In this review, we summarize the response to environmental stresses (hypoxic, oxidative, thermal, and mechanical stresses) in embryonic stem cells (ESCs) and their applications in the differentiation methods directed to specific lineages. Those stresses lead to activation of each specific transcription factor followed by the induction of downstream genes, and one of them regulates lineage specification. In short, hypoxic stress promotes the differentiation of ESCs to mesodermal lineages via HIF-1α activation. Concerning mechanical stress, high stiffness tends to promote mesodermal differentiation, while low stiffness promotes ectodermal differentiation via the modulation of YAP1. Furthermore, each step in the same lineage differentiation favors each appropriate stiffness of culture plate; for example, definitive endoderm favors high stiffness, while pancreatic progenitor favors low stiffness during pancreatic differentiation of human ESCs. Overall, treatments utilizing those stresses have no genotoxic or carcinogenic effects except oxidative stress; therefore, the differentiated cells are safe and could be useful for cell replacement therapy. In particular, the effect of mechanical stress on differentiation is becoming attractive for the field of regenerative medicine. Therefore, the development of a stress-mediated differentiation protocol is an important matter for the future.

## 1. Introduction

Embryonic stem cells (ESCs) and induced pluripotent stem cells (iPSCs) have a capacity to differentiate into any cell type [1,2,3] and are utilized for developmental research, disease modeling, drug development, and regenerative medicine. Presently, these pluripotent stem cells are revealed to have unique characteristics, such as pluripotency, naïve epigenetic state, and open chromatin (which means less condensed chromatin); the ratio between euchromatin and heterochromatin is also higher than it is in somatic cells [4]. This state allows transcriptional programs to switch their stem cell character rapidly upon induction of differentiation [4]. Therefore, these cells can have special functions such as infinite growth and pluripotency.

**Figure 1:**
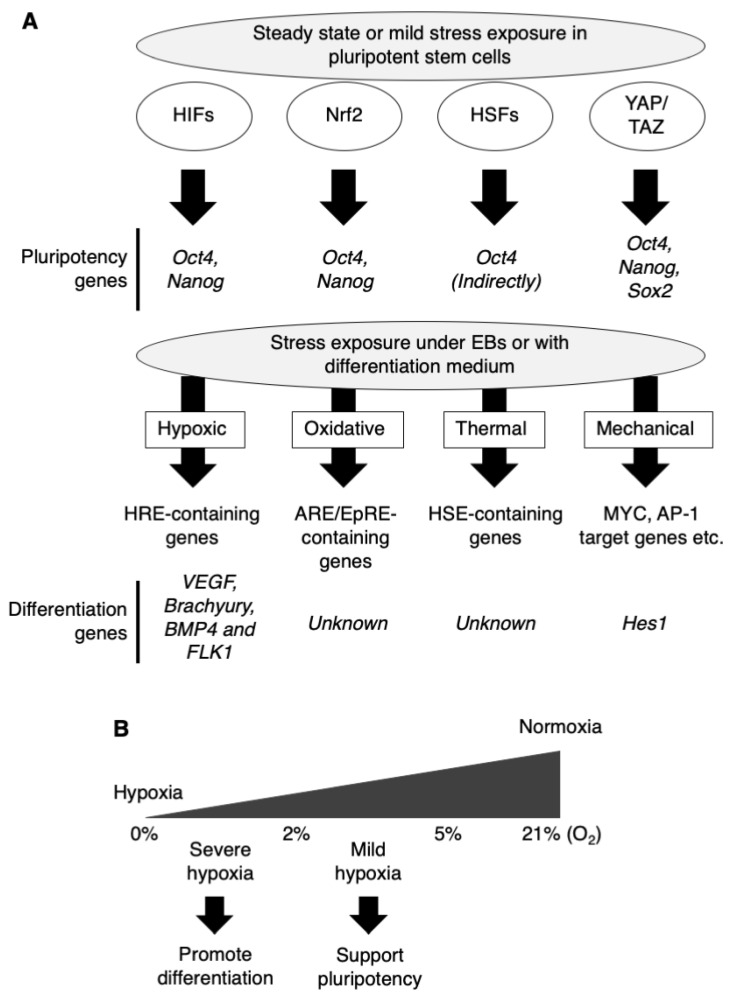
Environmental stresses and cellular response with transcriptional activation. (**A**) Steady-state levels of transcription factors as hypoxia-inducible factors (HIFs), Nrf2, HSFs, and YAP/TAZ involved in stress pathways support the maintenance of pluripotency in pluripotent stem cells independently of a stressor. Upon stress exposure, hypoxic stress leads to activation of HIFs (mainly HIF-1α and HIF-2α), followed by induction of hypoxia response element (HRE)-containing genes. Oxidative stress leads to activation of Nrf2, followed by induction of ARE/EpRE-containing genes. Thermal stress leads to activation of heat shock factors (HSFs) (mainly HSF1 and HSF2) followed by induction of HSE-containing genes. Mechanical stress leads to the activation of the YAP/TAZ transcriptional regulator of TEAD followed by induction of MYC and AP-1 target genes. In combination with embryoid body (EB) formation or differentiation medium, those stresses promote the differentiation to each specific lineage via activation of each target genes. EB, embryoid body; HRE, hypoxia response element; ARE, antioxidant-responsive element; EpRE, electrophile-responsive element; HSE, heat shock-responsive element. (**B**) About the intensity of hypoxic stress, mild hypoxia strengthens pluripotency in stem cell-maintaining medium, while severe hypoxia promote differentiation in differentiation medium.

Cells respond to a variety of stresses introduced by the environment to maintain their normal function, namely cellular homeostasis. These include hypoxic, oxidative, thermal, mechanical, physical, metabolic stress and others. In general, environmental stresses also include exposure to hormones, drugs, toxic substances, pollutants, and others; however, we focused only on stresses caused from extracellular space such as oxygen, temperature, and mechanical forces in this review. Such environmental stresses lead to dramatic cellular events through signal transduction and transcription of specific genes. In general, hypoxia activates hypoxia-inducible factor-1α (HIF-1α), and the resulting complex with HIF-1α binds to hypoxia response elements (HREs) and transcriptionally activates hundreds of genes involved in low oxygen adaptation [5,6,7]. In the case of oxidative stress, excess reactive oxygen species (ROS) and electrophiles activate NF-E2-related factor 2 (Nrf2), the complex with Jun and small Maf proteins binds to the antioxidant-responsive element (ARE) or the electrophile-response element (EpRE) and transcriptionally activates gene-coding antioxidant, antiapoptotic, metabolic, and detoxification proteins [8,9]. Elevated temperature activates heat shock factor 1 (HSF1), and it binds to the heat shock element (HSE) and transcriptionally activates genes encoding protein chaperones [10]. Furthermore, mechanical forces like extracellular stiffness lead to mechanotransduction via a remodeling of the cytoskeleton and activation of specific genes by YAP/TAZ transcriptional co-regulators, which bind primarily to enhancer elements by using TEAD factors as DNA-binding platforms [11,12]. YAP/TAZ-TEAD usually act in combination with other transcriptional factors (MYC, AP-1, etc.) bound at neighboring cis-regulatory elements [11]. Therefore, diverse downstream genes are regulated by those complexes. They target genes that promote cell proliferation, survival, and maintenance of stem cell fate [11,13]. All of these stress-inducible transcriptional factors are constantly expressed in pluripotent stem cells due to a low amount of each stress or other factors and may influence their proliferation, pluripotency and differentiation (Figure 1A). In fact, that stress pathway regulates the development of organs, as reviewed in some papers [14,15,16,17].

As mentioned above, pre-existing stress pathways support the maintenance of pluripotency via transcription of pluripotency genes, and mild stress exposure sometimes reinforces it. When a stressor is applied, those pathways are fully activated, and the direction and speed of differentiation are influenced (Figure 1A). Actually, some stress-inducible pathways are reported to enhance differentiation of pluripotent stem cells into specific lineages, as growth factors, hormones, culture environment, and 3D structures act on them. In this review, we summarize the response to such environmental stresses in pluripotent stem cells and its difference from somatic cells, and the differentiation protocol utilizing such stress responses. In general, these studies could be useful for developing differentiation protocols and elucidating the properties of stress response in pluripotent stem cells.

## 2. Hypoxic Stress for Directed Differentiation

Hypoxia is a consequence of a decrease in cellular oxygen. The ambient oxygen concentration is 21%. Hypoxic stress is defined as less than 5% of cases in which a molecular event in response to hypoxia is initiated [18,19]. When a cell is subjected to hypoxic stress, a cascade of hypoxic signaling is initiated through a family of transcription factors known as hypoxia-inducible factors (HIFs): HIF-1α, HIF-2α, and HIF-1β [18] (Figure 1A). During mouse embryogenesis, cellular O_2_ concentration is 1% to 5%, and this hypoxic state acts as a morphogen in many developmental systems via activation of HRE-containing genes [19]. Thus, the hypoxia–HIFs signal regulates organ development. Supporting this theory, HIF-1α deficient mice die by embryonic day 10.5 with cardiac malformations, vascular defects, and impaired erythropoiesis [20], showing that HIF-1α is critical for the development of such organs.

### 2.1. Involvement of Hypoxia Signaling in Pluripotency of Pluripotent Stem Cells

In pluripotent stem cells, HIFs have an important role in maintaining pluripotency and proliferation. Culturing human ESCs at a lower oxygen tension of 2–5% O_2_ is advantageous for their maintenance in terms of reduced spontaneous differentiation, improved proliferation and increased expression of key pluripotent markers [21,22,23,24,25,26]. In these functions of hypoxic signaling, HIF-2α is thought to be more predominant than HIF-1α, and HIF-2α was shown to directly regulate the expression of pluripotency genes *Oct4* and *Nanog* [24,27,28,29].

### 2.2. Effect of Hypoxic Stress on Directed Differentiation of Pluripotent Stem Cells

On the other hand, O_2_ tension, the partial pressure of O_2_, has been shown to regulate the embryonic development of organs, including the trachea, heart, lung, limb bud, and bone [14,30,31,32,33]. Recently, several reports have shown the utilization of modified O_2_ tension (hyperoxic or hypoxic stress) for the differentiation of pluripotent stem cells. High concentration of oxygen (more than 50%) is defined as hyperoxia and induces a cellular event inhibiting HIF-1α in the culture of endocrine progenitors [34,35,36]. In a previous study, we and some colleagues reported that a high oxygen condition (60% O_2_) improved the pancreatic differentiation via the inhibition of HIF-1α followed by repressed Notch-dependent gene *Hes1* expression [37]. *Hes1* represses *Ngn3*, an important factor for endocrine cell differentiation, by directly binding to this gene [38]. Additionally, severe hypoxia treatment (0.5–1% O_2_) during spontaneous differentiation in embryoid bodies (EBs) enhanced vascular-lineage differentiation [39] and mesoderm and cardiac differentiation [40] of mouse ESCs. Furthermore, cardiac and chondrogenic differentiation of human ESCs-derived EBs under 2–4% O_2_ was reported [41,42]. Hypoxic treatment (1% O_2_) also promotes the differentiation from mouse ESCs to arterial endothelial cells with endothelial differentiation medium [43] and 3% O_2_ promotes haemato-endothelial progenitor cells [44]. As a mechanism, hypoxia induces HIF-1α and regulates the expression of differentiation-guiding genes like *VEGF*, Cripto-1, and the genes involved in NOTCH1 signaling; it then promotes subsequent differentiation. In particular, Tsang et al. showed not only the usefulness of hypoxia treatment for the differentiation but also the machinery of the biphasic and sequential role of HIF-1α signaling in ESCs to arterial endothelial cells [43]. Initially, HIF-1α induces the transcription factor *Etv2* expression and then enhances the generation of endothelial cell progenitors; then, HIF-1α induces *Dll4* expression and activates NOTCH1 signaling, resulting in the maturation of their progenitors to an arterial endothelial cell fate. Thus, NOTCH1 signaling is supposed to be a key factor for HIF-1α-mediated differentiation. Recently, it was shown that a mild hypoxic condition (10% O_2_) promoted hepatocyte differentiation in liver buds from human iPSCs in combination with organoid technology [45]. They concluded that the inhibition of the transforming growth factor beta (TGFB) signal was involved in this effect because TGFB isoforms are known to affect fetal hepatocyte development [46]. From the above reports, hypoxic treatment cannot be a start switch for the differentiation, but can act like a handle to direct differentiating cells at specific lineages. In addition, interestingly, severe hypoxic treatment (less than 5% O_2_) promotes differentiation while mild hypoxic treatments (more than 5%) are used for strengthening pluripotency (Figure 1B). We summarized these reports in Table 1.

According to the above reports, hypoxic stress promotes the differentiation to mesodermal lineages and sometimes endodermal lineages. Another treatment for chemical hypoxia, such as CoCl_2_ and deferoxamine, could be useful for such vascular, cardiac, pancreatic, and hepatic differentiation of pluripotent stem cells, because such treatments stabilize HIF-1α via inhibition of prolyl hydroxylase domain proteins (PHDs). CoCl_2_ is easily used by adding it to regular cell culture media at a final concentration of 100 μM and incubating the cultures for 24 h in a conventional incubator.

## 3. Oxidative Stress for Directed Differentiation

Oxidative stress caused by reactive oxygen species (ROS) was initially presumed to cause cell damage and apoptotic cell death. They are now recognized as important molecules that regulate many cell signaling and biological processes, such as activation of transcription factors, induction of defense genes, phosphorylation of kinases, and mobilization of ions in transport systems [61,62]. 

### 3.1. Involvement of Oxidative Stress Signaling in Pluripotency of Pluripotent Stem Cells

Oxidative stress signaling is also an essential process in pluripotent stem cells. In fact, the genetic stability of ESCs requires moderate levels of ROS expression [63,64]. Surprisingly, Li and Marban showed that the addition of high-dose antioxidant to the medium of human ESCs increases aneuploidy, suggesting that physiological levels of intracellular ROS are required for the DNA repair pathway to maintain genomic stability [64]. Pluripotent stem cells are sensitive to excess oxidative stress; however, Guo et al. reported that mouse ESCs are resistant to oxidative-stress-induced senescence compared to differentiated cells, but not to oxidative stress-induced apoptosis [65]. They suggested that ESCs might have unique mechanisms to protect self-renewal capacity against such stress. In the case of multipotent stem cells, low levels of ROS are reported to enhance mesenchymal stem cells (MSCs) proliferation and migration through the activation of extracellular-signal-regulated kinases (ERK) 1/2 and Jun-1/2 pathways [63]. Thus, moderate levels of ROS are key molecules to maintain the potency in both pluripotent and multipotent stem cells.

### 3.2. Effect of Oxidative Stress on Directed Differentiation of Pluripotent Stem Cells

Concerning the effect of oxidative stress on differentiation, treatment with oxidizing agent paraquat, which induces cellular ROS followed by oxidative stress, leads to the spontaneous differentiation to neuronal cells of human ESCs via suppressed expression of stemness genes and enhanced expression of neuronal differentiation markers PAX6, NEUROD1, HOXA1, NCAM, GFRA1, and TUJ1 [47]. H_2_O_2_ treatment induced a similar effect, in which the activation of MAPK-ERK1/2 pathways was shown to be involved [47]. In addition, treatment with buthionine sulfoximine, which inhibits glutathione, induces ROS and causes oxidative stress in human ESCs because glutathione usually reduces H_2_O_2_ with catalysis by glutathione peroxidase. This treatment promoted the differentiation of human ESCs towards mesendodermal lineages with enhanced expression of mesodermal genes T and *MYOG* and endodermal genes *HNF3B* and *SOX17* [48,63]. Furthermore, ROS production by icariin or by NADPH oxidase-4 (NOX-4) promotes ESC differentiation into cardiomyocytes [49,50]. For all these effects, MAPK-ERK1/2 pathways are shown to be activated by oxidative stress and such activation leads to differentiation. Nrf2 is known to control self-renewal and pluripotency in human ESCs as described elsewhere [66], and additionally, Jang et al. reported an interesting machinery, namely that the primary cilium, which is a microtubule-based organelle, and autophagy-Nrf2 control axis decide cell fate to neuroectoderm in human ESCs [51]. They defined Nrf2-binding site in *OCT4* and *NANOG* promoter and showed that Nrf2 directly regulates their expressions. In addition, the differentiation potential of each iPSC line to neuroectoderm can be predicted by the levels of Nrf2 expression and the suppression of Nrf2 in the iPSC line, which has poor differentiation potential to rescue the differentiation to neural fate, supporting the key role of Nrf2 in early lineage determination [51]. If this is true, the suppression of Nrf2 is a useful method for neural differentiation of human iPSCs. We summarized these reports in Table 1.

## 4. Thermal Stress for Directed Differentiation

Elevated temperature (generally exceeding 40 °C in cultured cells) causes denaturation of proteins and leads to protein aggregation, which results in cellular toxicity and cell death [67]. To prevent such a crisis, heat shock factor 1 (HSF1) is activated upon cell stress and stimulates transcription of genes encoding molecular chaperones [68]. HSF1 is constitutively expressed in most tissues and cell types and appears to be regulated primarily through protein–protein interactions and posttranslational mechanisms [69,70]. In the absence of stress, the DNA-binding activity of HSF1 is repressed through the interaction with chaperones such as HSP70, HSP90, TRiC, and others, and the majority of HSF1 exists in an inert monomeric form [70,71,72,73]. Upon heat stress, the influx of misfolded proteins prevents chaperones from binding to HSF1 monomers, and this leads to the de-repression of HSF1 from chaperones, followed by conversion of monomer to DNA binding-competent trimers [71,72,73]. Then HSF1, transcriptionally activates genes encoding molecular chaperones, which are essential for protein folding, preventing misfolding and restoring the native conformation of misfolded proteins, and components of the ubiquitin proteasome system [68]. The coordinated action of these protein quality-control genes restores protein homeostasis when it is disrupted by heat shock [68,74]. In almost all cells, this pathway is essential for protein folding of denatured proteins, which is called heat shock response (HSR). 

### 4.1. Involvement of Thermal Stress Signaling in Pluripotency of Pluripotent Stem Cells

In pluripotent stem cells, HSR normally occurs, and it was shown that human and mouse ESCs are more resistant against heat stress than differentiated cells [75]. Global protein synthesis in ESCs is enhanced to maintain its pluripotency, showing the huge amount of proteins constantly produced and utilized for cell life [76]. Accordingly, human pluripotent stem cells exhibit enhanced assembly of the TRiC/CCT complex, which is a chaperonin that facilitates the folding of proteins [77]. Thus, it is assumed that pluripotent stem cells might have a higher chaperon function than somatic cells to prevent protein-aggregation-induced toxicity. Furthermore, it was reported that thermal stress (42 °C) changes the expression of hundreds of genes via the activation of decommissioning of their enhancers mediated by not only HSF1 and AP-1, but also pluripotency factors such as NANOG, KLF4, and OCT4 [78].

### 4.2. Effect of Thermal Stress on Directed Differentiation of Pluripotent Stem Cells

Concerning the utilization of thermal stress signaling, Byun et al. reported that heat shock treatment (46 °C) caused differentiation of human ESCs via repression of *OCT4* expression by HSF1 [79]. In this report, they showed that HSF1 negatively regulates *OCT4* expression and SAPK/JNK mediates its effect via phosphorylation. In addition, Koga et al. reported unique research in which mild electrical stimulation (1 V/cm, 55 pps) with heat shock (42 °C) facilitated the differentiation of mouse ESCs to definitive endoderm, with an upregulation of heat shock protein 72 [52]. Those reports are summarized in Table 1.

## 5. Mechanical Stress for Directed Differentiation

Mechanical forces have been revealed to regulate many physiological process of the cells [80,81,82]. There are many types of mechanical forces, such as tension, compression, pressure, and shear [81]. The properties of a material as stiffness, compliance, elasticity, and rigidity also affect mechanotransduction in the cells [81]. Overall, the response to mechanical forces is known to regulate cell growth, differentiation, shape changes, and cell death [82].

### 5.1. Involvement of Mechanical Stress Signaling in Pluripotency of Pluripotent Stem Cells

There are two mechanical forces involved in stem cell function and differentiation: fluid shear stress and a signal from the stiffness of the culture environment. Theoretically, cells sense a mechanical environment mainly via the actin cytoskeleton tension and integrin-mediated focal adhesion, which interact with external biophysical stimuli to elicit downstream signaling (mechanotransductive signaling) [80,81,82]. In addition to those two pathways, pluripotent stem cells also use mechanosensitive ion channels Piezo1 and their primary cilium to regulate mechanotransduction [83,84]. In fact, mechanical signals promote osteogenic fate through a primary cilia-mediated mechanism [85]. Recently, it was shown that stiff substrate leads to engagement of integrins and activates focal adhesions as focal adhesion kinase (FAK) and steroid receptor coactivator (SRC). Then, FAK phosphorylates and activates YAP, leading to the activation and nuclear translocation of YAP/TAZ transcription factor, which is known to be involved in such cellular mechanoresponses [13,86]. In pluripotent stem cells, it was shown that YAP binds to promoters of pluripotent genes and is required for the pluripotency of mouse ESCs [87].

### 5.2. Effect of Mechanical Stress on Directed Differentiation of Pluripotent Stem Cells

Fluid shear stress in endothelial cells is a physical force from flowing blood in the vasculature [88]. It has major effects on vascular development and function. Previously, fluid shear stress has been shown to induce the vascular endothelial cell differentiation of ESCs via tyrosine phosphorylation of Flk-1 [53]. This stress also promoted endothelial and hematopoietic differentiation of ESCs via Flk1 activation [54]. In addition, Adamo et al. showed that fluid shear stress increases the expression of Runx1 in the hematopoietic progenitor cells differentiated from ESCs, concomitantly augmenting their hematopoietic colony-forming potential [55].

Another type of exogenous mechanical force could affect the differentiation of pluripotent stem cells to several cell lineages. Tissues have a variation of stiffness [17], known as hard and soft tissues. Thus, stiffness of culture plate and extracellular matrix (ECM) affect the cell fate of differentiation, because each tissue has unique elasticity, as explained above [89]. Therefore, the differentiation potentials of stem cells to distinct lineages could be improved if the cells are cultured in the mechanical microenvironment mimicking their tissue elasticity in vivo [17,90,91]. Regarding the differentiation method considering such stiffness and elasticity, mechanical stress led to *Oct4* gene downregulation in mouse ESCs, showing that small forces might play important roles in the early development of soft embryos [92]. In addition, it was reported that an increase in capsule stiffness enhanced differentiation of human ESCs to definitive endoderm via an increase in pSMAD/pAkt levels, while suppressing differentiation to pancreatic progenitor [56]. Another group has shown that decreased stiffness of capsule enhanced endodermal differentiation of mouse ESCs [59]. Others reported that mesodermal differentiation was upregulated when stiffness increased on fibronectin-coated polydimethylsiloxane (PDMS) substrate [57]. Interestingly, Zoldan et al. compared the effect of scaffold elasticity on the differentiation to three germ layers of human ESCs and showed that high elasticity promoted mesodermal, intermediate-elasticity endodermal, and low-elasticity ectodermal differentiation [58]. These reports suggest an implication of tissue-specific stiffness to such elasticity-dependent differentiation; however, the precise mechanism has not been elucidated. Furthermore, cell confinement was recently revealed to increase high PDX1-expressing cells differentiated from human ESCs, suggesting that loss of YAP1 expression was involved in cell-confinement-induced differentiation to pancreatic progenitors [60]. We summarized these reports in Table 1.

## 6. Physical Stimulation for Directed Differentiation

### 6.1. Involvement of Physical Stimulation in Pluripotency of Pluripotent Stem Cells

There are other unique environmental stimuli such as microgravity and the electromagnetic effect. Up to the present, research about the effect of space flight on organ development is increasing with the use of pluripotent stem cells [93]. Regarding pluripotency, the effect of space microgravity on the self-renewal capacity of mouse iPSCs was studied via live-imaging of *Oct4*-GFP reporter in spacecraft [94]. In microgravity condition, cells in iPSC clones spread out more rapidly than those in ground 1 g condition and easily recovered *Oct4* expression, suggesting that microgravity leads to more dynamic behavior of iPSCs, even while they maintain pluripotency [94]. Furthermore, Blaber et al. showed that exposure to microgravity inhibited mouse ESCs differentiation in embryoid bodies and cells recovered from microgravity-unloaded embryoid bodies showed greater stemness, indicating that the condition of microgravity maintains their pluripotency [95]. Generally, microgravity seems to maintain pluripotency than ground gravity without the signals directing to differentiation.

### 6.2. Effect of Physical Stimuli on Directed Differentiation of Pluripotent Stem Cells

Regarding the utilization of physical stimulation for stem cell differentiation, Lei et al. showed that rotary suspension culture, which gives a microgravity to cells, promoted mesodermal differentiation of mouse ESCs via Wnt/β-catenin pathway, while this protocol repressed ectodermal differentiation [96,97]. Additionally, Li et al. reported that microgravity promoted myocardial differentiation of mouse iPSCs, which was shown by down-regulation of *Oct4* reporter and upregulation of α-myosin heavy chain reporter via time-lapse imaging of the cells in a bioreactor during space flight [98]. Furthermore, another group reported that microgravity and 3D culture enhanced the differentiation of cardiac progenitor from human ESCs and iPSCs with the production of enriched cardiomyocytes (99% purity) and high viability [99]. Regarding endodermal differentiation, the culturing embryoid bodies were placed in a rotary bioreactor, which simulates microgravity, and upregulated all of the definitive endoderm markers as *Foxa2* and *Sox17* in mouse ESCs, indicating that this biophysical stimulation enhanced directed endodermal differentiation [100]. A rotating bioreactor with a biodegradable polymer scaffold also can yield functional and transplantable hepatocyte from mouse ESCs [101]. Another stimuli to consider is the electromagnetic field (EMF) environment, which is reported to regulate cell fate conversion of several types of stem cells [102]. It was reported that extremely low-frequency EMF (50 Hz, 1 mT), which is usually generated from power lines and household electric appliances, promoted neuronal differentiation of neural stem cells via up-regulation of TRPC1 expression [103]. Additionally, Huang et al. reported that a pulsed EMF with magnetic nanoparticle composite scaffold induced osteogenic differentiation of bone marrow mesenchymal stem cells [104]. Regarding the mechanism of the effect of EMF, it was reported that single electrical field pulse of 500 V/m promoted cardiomyocyte differentiation of mouse ESCs via intracellular ROS generation and nuclear factor κB (NF-κB) activation [105]. Furthermore, treatment of differentiating mouse ESCs with static EMF (0.4–2 mT) was reported to stimulate vasculogenesis and chondro-osteogenesis via ROS generation and vascular endothelial growth factor (VEGF) induction which is regulated by ERK1/2 [106]. The protocol mentioned above is summarized in Table 2. 

## 7. Other Candidates for the Method of Directed Differentiation with Environmental Stresses

**Figure 2:**
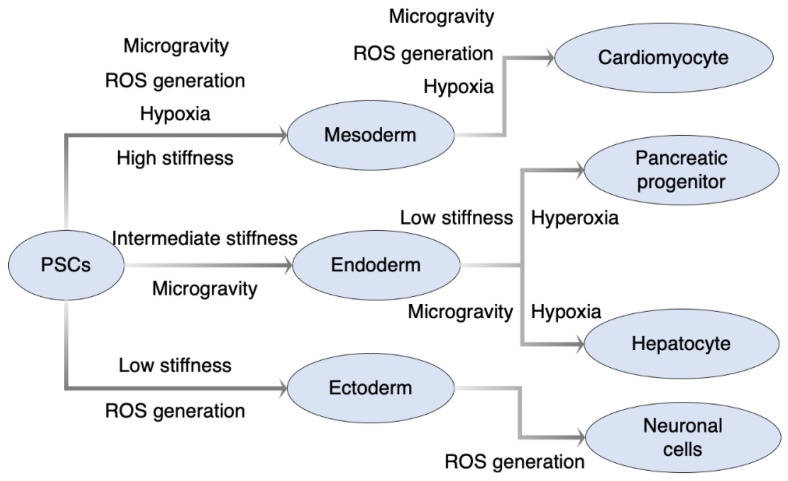
Summary of directed differentiation with environmental stresses. Hypoxia treatment tends to promote mesodermal differentiation followed by mesoderm-derived lineages like cardiomyocytes. ROS generation by ROS-inducing agents as paraquat, icariin, and H_2_O_2_, and treatment as EMF could also promote both mesodermal and ectodermal differentiation. Regarding mechanical stress, there is suitable stiffness of the culture substrate to the differentiation to each 3 germ layers, and it is also utilized for further differentiation like pancreatic progenitors from definitive endoderm. PSCs, pluripotent stem cells; ROS, reactive oxygen species; EMF, electromagnetic field.

As mentioned above, there are many reports about the utilization of environmental stresses and physical stimulation for the directed differentiation to specific lineages of human and mouse pluripotent stem cells (Figure 2). Generally, hypoxia treatment is more apt to direct those stem cells toward the mesoderm and its derived lineages. ROS generation via oxidative stress-inducing chemicals or treatments promotes the differentiation to mesodermal and ectodermal lineages. The stiffness of culture substrate is influential in the differentiation to each 3 germ layers, and could be more important in the differentiation to endoderm and its derived lineages, especially the pancreas. Simulated microgravity seems to promote both mesodermal and endodermal lineages with each defined differentiation media. In addition to type and intensity of the stress, the timing of stress exposure is also important and should be considered to direct differentiating progenitors to its mature tissues. The relationship between capsule stiffness and each stage of pancreatic differentiation was well studied by Richardson et al. [56]. However, which timing of stress exposure is effective on each stage of the process of differentiating cells to other lineages has not been fully studied throughout all stress types. Furthermore, any protocol with the combination of multiple environmental stresses for the directed differentiation has not been reported. Recently, 3D culture and organoid formation were revealed to be effective in getting mature tissues such as cerebral cortex, liver bud, pancreatic islet, and others [107,108,109]. The combination of stress exposure and such 3D culture technology seems to be a more powerful tool for the directed differentiation at least to the above tissues. These could be attractive research fields for developmental biology and regenerative medicine.

As with other types of stresses, metabolic stress (overnutrition or starvation) and environmental pH are also reported to be utilized for stem cell technology. Starvation with single-cell plating enhanced transfection efficiency of siRNAs and plasmids into human ESCs [110]. In addition, intracellular pH influences proliferation and differentiation of pluripotent stem cells [111]. Inhibition of Na^+^/H^+^ exchanger 1 (NHE1), which plays a key role in intracellular pH regulation, prevents cardiomyocyte differentiation of mouse ESCs, while increased expression of NHE1 facilitates that differentiation [112]. Ulmschneider et al. also reported that intracellular pH increased with differentiation of mouse ESCs and when prevented, attenuated spontaneous differentiation of naïve cells [113]. Furthermore, Kim et al. examined the effect of medium pH on the differentiation of mouse ESCs and showed that the high pH level of 7.8 enhanced mesendodermal differentiation [114].

## 8. Conclusions

Environmental stimuli, such as hypoxic, thermal, mechanical, and physical stimuli, are not especially harmful and do not have genotoxic or carcinogenic effects except oxidative stress. Therefore, they are relatively safe compared to artificial chemicals and gene transfer for the differentiation of clinical-grade cells and organoids. In particular, mechanical forces could be infinitely modified by a variation of materials and coated ECM for culture plates and could be an attractive research field for regenerative medicine. In addition, the effect of exposure to gravity and the EMF on the differentiation of pluripotent stem cells has not been fully studied; therefore, those are promising research areas for the development of the differentiation method via physical stimulation.

## Figures and Tables

**Table 1:** The differentiation protocol of pluripotent stem cells using a stress response pathway.

Stress	Cell Type	Directed Cell Type	Mechanism	References
**Hypoxic stress**				
Hyperoxia (60% O_2_)	Mouse ESCs ^1^, human iPSCs ^2^	Pancreatic beta cell	Inhibition of HIF-1αfollowed by Hes1 repression	[37]
Hypoxia (1% O_2_)	Mouse ESCs	Vascular lineage	HIF-1-mediated inverse regulation of Oct4 (down) and VEGF (up)	[39]
Hypoxia (0.5% O_2_)	Mouse ESCs	Mesoderm and cardiomyocyte	HIF-1α mediated Cripto-1 expression	[40]
Hypoxia (2% O_2_)	Human ESCs	Chondrocyte	Undescribed	[42]
Hypoxia (1% O_2_)	Mouse ESCs	Arterial endothelial cells	Activation of ETV2 and NOTCH1 signaling by HIF-1α	[43]
Hypoxia (4% O_2_)	Human ESCs	Cardiomyocytes	Undescribed	[41]
Hypoxia (3% O_2_)	Mouse ESCs	Mesoderm and hemangioblast	Accelerated expression of Brachyury, BMP4 and FLK1 via Arnt	[44]
Mild hypoxia (10% O_2_)	Human iPSCs	Hepatocyte	TGFB signal inhibition	[45]
**Oxidative stress**				
Paraquat (25 µM)	Human ESCs	Neuronal cells	ROS ^3^ and activation of MAPK-ERK1/2	[47]
Buthionine sulfoximine (0.2 mM)	Human ESCs	Mesodermal and endodermal lineages	Inactivation of p38 and AKT as well as concomitant transient increase in JNK and ERK signaling	[48]
Icariin	Mouse ESCs	Cardiomyocyte	ROS generation and the subsequent activation of p38 MAPK	[49]
H_2_O_2_ (1~100 nM)	Mouse ESCs	Cardiomyocyte	p38 activation and MEF2C nuclear translocation	[50]
Nrf2 shRNA	Human iPSCs	Neuroectoderm	Suppression of Nrf2 binding to pluripotency genes OCT4 and NANOG	[51]
**Thermal stress**				
Heat shock with mild electrical stimulation (42 °C, 55 pps)	Mouse ESCs	Pdx1-expressing pancreatic progenitors from definitive endoderm	Upregulation of Hsp72 and activation of Akt, ERK, p38 and JNK (putative).	[52]
**Mechanical stress**				
Fluid shear stress	Mouse ESCs	Vascular endothelial cell	Flk-1 activation and VEGF production	[53]
Fluid shear stress	Mouse ESCs	Endothelial and hematopoietic cell	Flk1 activation	[54]
Fluid shear stress	Mouse ESCs	Hematopoietic cell	Increased Runx1 expression	[55]
High stiffness (BAlg ^4^ capsule, ~22 kPa)	Human ESCs	Definitive endoderm	Increase in pSMAD/pAkt	[56]
Low stiffness (BAlg capsule, ~4 kPa)	Human ESCs	Pancreatic progenitor	Decrease in SHH signaling	[56]
High stiffness	Human ESCs	Mesoderm	Undescribed	[57]
High stiffness (3D scaffold, 1.5–6 MPa)	Human ESCs	Mesoderm	Undescribed (similar elasticity during gastrulation could be related)	[58]
Intermediate stiffness (3D scaffold, 0.1–1 MPa)	Human ESCs	Endoderm	Undescribed (similar elasticity during gastrulation could be related)	[58]
Low stiffness (3D scaffold, <0.1 MPa)	Human ESCs	Ectoderm	Undescribed (similar elasticity during gastrulation could be related)	[58]
Low stiffness (encapsulated by alginate microbeads)	Mouse ESCs	Endoderm	Undescribed	[59]
Confinement (~300 µm^2^)	Human ESCs	Pancreatic endocrine progenitor	Inhibition of YAP1	[60]

^1^ ESCs, embryonic stem cells; ^2^ iPSCs, induced pluripotent stem cells; ^3^ ROS, reactive oxygen species; ^4^ BAlg, barium alginate.

**Table 2:** The differentiation protocol of pluripotent stem cells using physical stimuli.

Stress	Cell Type	Directed Cell Type	Mechanism	References
**Microgravity**				
Rotary suspension culture	Mouse ESCs ^1^	Mesoderm	Enhancement of Wnt/β-catenin signaling	[96,97]
Spaceflight	Mouse iPSCs ^2^	Cardiomyocyte	Undescribed	[98]
Simulated microgravity and 3D culture	Human ESCs and iPSCs	Cardiomyocyte	Increased proliferation and viability of cardiac progenitors via up-regulation of heat shock proteins and *BIRC5*	[99]
Simulated microgravity in rotary bioreactor	Mouse ESCs (embryoid body)	Definitive endoderm	Undescribed	[100]
Simulated microgravity in rotary bioreactor	Mouse ESCs	Hepatocyte	Undescribed	[101]
**EMF** ^3^				
Single electrical field (500 V/m)	Mouse ESCs	Cardiomyocyte	Intracellular ROS ^4^ generation and NF-κB ^5^ activation	[105]
Static EMF (0.4–2 mT)	Mouse ESCs	Vasculogenesis and chondro-osteogenesis	Intracellular ROS generation and VEGF ^6^ induction	[106]

^1^ ESCs, embryonic stem cells; ^2^ iPSCs, induced pluripotent stem cells; ^3^ EMF, electromagnetic field; ^4^ ROS, reactive oxygen species; ^5^ NF-κB, nuclear factor κB; ^6^ VEGF, vascular endothelial growth factor.

## Data Availability

Not applicable.
